# Supplementary material for: Probabilistic greedy algorithm solver using magnetic tunneling junctions for traveling salesman problem
Source: Nat Commun. 2025 Dec 4;17:189. doi: 10.1038/s41467-025-66864-9 (PMC12780240; doi:10.1038/s41467-025-66864-9)
Supplement: Supplementary file 1 — Supplementary Information [file 41467_2025_66864_MOESM1_ESM.pdf]

Supplementary Information

Probabilistic Greedy Algorithm Solver Using Magnetic Tunneling Junctions for Traveling Salesman Problem

Ran Zhang[1], Xiaohan Li[1], Caihua Wan[1, 2, 6, *], Raik Hoffmann[3], Meike Hindenberg[3], Yingqian Xu[1], Shiqiang Liu[1], Dehao Kong[1], Shilong Xiong[1], Shikun He[4], Alptekin Vardar[3], Qiang Dai[4], Junlu Gong[4], Yihui Sun[4], Zejie Zheng[4], Thomas Kämpfe[3, 5, *], Guoqiang Yu[1, 2, 6] and Xiufeng Han[1, 2, 6, *]

[1]Beijing National Laboratory for Condensed Matter Physics, Institute of Physics, University of Chinese Academy of Sciences, Chinese Academy of Sciences, Beijing 100190, China

[2]Center of Materials Science and Optoelectronics Engineering, University of Chinese Academy of Sciences, Beijing 100049, China

[3]Fraunhofer IPMS, Center Nanoelectronic Technologies, 01109 Dresden, Germany

[4]Zhejiang Hikstor Technology Co. Ltd, Hangzhou 311305, China

[5]TU Braunschweig, Institute for CMOS Design, 38106 Braunschweig, Germany

[6]Songshan Lake Materials Laboratory, Dongguan, Guangdong 523808, China

E-Mail: thomas.kaempfe@ipms.fraunhofer.de ; wancaihua@iphy.ac.cn ; xfhan@iphy.ac.cn

Supplement I: Measurement and program-controlled system description

The setup used for testing and characterizing spin-transfer torque magnetic tunneling junction (STT-MTJ) devices combines precise electrical measurements and multi-device control, integrating hardware and software components to ensure fast, accurate, and reliable data acquisition.

Hardware configuration

The MTJ samples are arranged on a silicon die with a structure comprising from top to bottom capping/CoFeB/Mo/CoFeB/MgO/CoFeB/Mo/$[Co/Pt]_n$-based synthetic anti-ferromagnetic layers. These samples connect to the measurement system through a high-precision probe card with a 100 μm pitch and 25 individual pins, ensuring minimal contact resistance and high reproducibility. Signals from the probe card are routed via SMB-Dupont micro coaxial cables to an adapter board, which serves as a signal conditioning interface. The adapter board connects to SHDB62M-DB62M-LL type DB62 cables, known for shielding properties that reduce interference, ensuring the accurate transfer of signals to the high-resolution measurement instruments within the NI PXIe system.

The instrumentation consists of a source measure unit (SMU) and a pattern generator module integrated into the NI PXIe system. The NI PXIe-4163 SMU has 24 independent channels, each capable of delivering up to ±10 V and measuring currents down to nanoampere levels, allowing for precise sourcing and measurement operations. The pattern generator applies voltage or current pulses to induce switching in the magnetic free layer of the MTJ, enabling the study of dynamic stochastic behavior. The pulse duration and intervals are controlled with high precision to capture time-sensitive switching events.

Software and control system

The Python-based control program acts as the central coordinator of the experimental process, interfacing with the NI PXIe system to manage data acquisition and device control. The program employs a continuous loop structure where each iteration involves reading the current state of the MTJ devices, processing the data according to a specified algorithm, and then adjusting the excitation voltage for the next step.

Data acquisition is initiated through the SMU module, which measures the resistance states of the MTJs after each applied voltage pulse. The Python program uses the NI-DAQmx driver to communicate with the SMU, retrieving measurements as arrays of resistance values that are timestamped and stored for analysis. Once the data are read, they undergo a filtering process to remove any electrical noise and ensure data integrity.

Based on the processed resistance data, the program calculates the corresponding switching probability using statistical methods. The feedback mechanism uses a predefined mathematical relationship between the switching probability and the applied voltage, which is typically modeled as a sigmoidal function. The program determines the required voltage directly from this relationship, calculating it based on the desired switching probability for the next iteration. This voltage is then applied to the MTJ in the next step, ensuring a precise adjustment that aligns with the defined model rather than relying on observed values to make incremental changes.

This approach enables real-time tuning of the MTJ excitation voltage based on a deterministic relationship between probability and voltage. This ensures consistency across measurements. The algorithm's output is directly mapped to the necessary voltage adjustments, allowing for continuous adaptation to maintain the target probabilistic behavior.

Data acquisition and processing

The adaptive control process involves a real-time loop where data acquisition, processing, and voltage adjustment occur continuously. After reading the resistance data from the SMU, the Python program applies statistical algorithms to calculate the switching probability for each device.

In each iteration, the desired switching probability serves as input to the model, which outputs the voltage that should be applied in the next step. This calculated voltage is set on the pattern generator, which updates the excitation conditions for the MTJ accordingly. The entire process repeats for each iteration, ensuring that the MTJ maintains the desired stochastic characteristics by precisely adjusting the applied voltage as determined by the established sigmoid relationship. The use of a defined mathematical model for determining the excitation voltage ensures that adjustments are both accurate and predictable. This method enables the algorithm to fine-tune the random number generation process effectively, making it suitable for applications in probabilistic computing and stochastic optimization.

System integration and communication

The components of the measurement system are tightly integrated for efficient communication and data exchange. The NI-DAQmx drivers facilitate interaction with the NI PXIe system, allowing for synchronized operations such as simultaneous pulse application and resistance measurement. Measurement data is timestamped and stored in a structured database, ensuring traceability and enabling systematic investigation of the effects of various experimental parameters on switching behavior.

This setup for characterizing MTJ-based true random number generators provides high-precision control over electrical stimuli and supports adaptive testing with real-time feedback. The integration of the NI PXIe system, high-speed data acquisition, and Python-based software creates a versatile and efficient platform, forming a strong foundation for exploring MTJ-based optimization frameworks and applications in probabilistic computing.

Supplement II: Bayesian inference network for transforming Bernoulli random numbers into arbitrary probability distributions

This section describes a method for converting basic Bernoulli random numbers into random numbers that follow arbitrary probability distribution functions (PDFs) using a Bayesian inference network. The approach enables the generation of random numbers with specified statistical properties directly at the hardware level, which is highly beneficial for applications in stochastic computing, probabilistic algorithms, and hardware-accelerated optimization tasks. The technique leverages the tunable switching probability of magnetic tunnel junctions (MTJs) to implement a multi-bit true random number generator (TRNG) that can produce random outputs following a desired PDF.

Concept Overview

The fundamental building block for this approach is a Bernoulli random number generator, which produces binary outcomes (0 or 1) with a probability p of switching to '1' and $1-p$ of switching to '0'. By configuring the switching probability p of the MTJs through a controlled voltage, these Bernoulli random numbers can be generated with high precision. However, many applications require random numbers with more complex distributions than a simple Bernoulli process can provide.

To transform these basic Bernoulli outputs into multi-bit random numbers that follow a specific PDF, a Bayesian network is utilized. The network encodes the relationships between a series of binary random variables, allowing the construction of higher-resolution random numbers with controlled statistical properties.

Bayesian Network Structure

The Bayesian network used for this transformation is organized in layers, where each layer corresponds to a bit in the final output random number. For example, a 4-bit random number generator would have four layers in the network, with each layer corresponding to one binary digit. The nodes in each layer represent binary random variables whose output probabilities depend on the states of the nodes in the preceding layers.

Although the transformation from Bernoulli-distributed outputs to arbitrary PDFs involves conditional probability logic, in our implementation this process is executed in a pipelined, clock-synchronized sequence. The necessary control parameters—such as MTJ pulse width or voltage amplitude—are pre-computed based on the desired PDF and dynamically applied without real-time feedback delay. As verified in [Zhang et al., Phys. Rev. Applied, 2025], this

structure supports high-speed operation using only digital logic, ensuring that conditional probability logic does not become a bottleneck in MTJ-based TRNG systems.

The conditional dependencies between these nodes are defined by a conditional probability table (CPT), which specifies the probability of each node's output (0 or 1) given the states of the parent nodes. The CPT serves as the core mechanism that allows the network to shape the distribution of the final multi-bit output according to the desired PDF.

Algorithm for Transformation from Bernoulli to Arbitrary Distributions

The algorithm for generating random numbers with a specified PDF using the Bayesian network proceeds as follows:

1. **Initialization of the Network:**
    - The network consists of multiple layers of nodes, where each node represents a bit in the output random number. For an *n*-bit generator, there are *n* layers in the network.
    - Each node's output is influenced by the preceding nodes through directed edges, with the probability of each node's state determined by the CPT.
2. **Setting the Probability for the First Node (Most Significant Bit):**
    - The first node (corresponding to the most significant bit) is initialized with a switching probability p that directly maps to a portion of the target PDF. This probability is determined based on the cumulative distribution function (CDF) of the desired PDF.
    - The voltage applied to the MTJ representing the first node is set according to the inverse relationship between the switching probability and voltage, ensuring that the MTJ's output reflects the specified probability.
3. **Conditional Probability Adjustment for Subsequent Nodes:**
    - For each subsequent node, the switching probability is adjusted based on the states of all previous nodes. The CPT provides the conditional probabilities for the current node, considering the binary values of the parent nodes.
    - The switching probability for each node is calculated as: $p_i = \frac{\sum_{N \in S_i} P(N)}{\sum_{N \in S_{i-1}} P(N)}$,

      where S_i represents the set of possible output values for the network given the states of the previous nodes, and $P(N)$ is the probability from the desired distribution.

- The appropriate voltage for the MTJ at this layer is then set using the calculated conditional probability and the corresponding inverse switching function.

4. **Cascading the Random Number Generation:**
   - The process continues sequentially through all layers of the network. The output of each node determines the switching conditions for the following nodes, effectively "cascading" the random number generation process.
   - The nonvolatile nature of the MTJs ensures that the state of each node can be maintained until the final output random number is fully determined.
5. **Final Output Generation:**
   - After all nodes in the network have been evaluated, the final random number is constructed from the outputs of each node. The resulting multi-bit number follows the specified probability distribution.

Example: Generating a 4-Bit Random Number with a Specified PDF

To illustrate the process, consider a 4-bit TRNG designed to produce random numbers from 0 to 15 with a given PDF $P(N)$. The network consists of four layers (nodes A, B, C, and D), corresponding to the four bits of the output.

1. **Determine p_A for Node A (Most Significant Bit):**
   - Let p_A be the probability of node A switching to '1'. This is calculated based on the cumulative probabilities for outputs 8 to 15: $P_A = \sum_{N=8}^{15} P(N)$.
   - The voltage for node A is set to achieve this switching probability.
2. **Calculate $p_B|A$ for Node B:**
   - The probability for node B depends on the output of node A:
     - If A=1, then: $p_B \mid (\text{A=1}) = \dfrac{\sum_{N=12}^{15} P(N)}{\sum_{N=8}^{15} P(N)}$.
     - If A=0, then: $p_B \mid (\text{A=0}) = \dfrac{\sum_{N=4}^{7} P(N)}{\sum_{N=0}^{7} P(N)}$.
   - The appropriate voltage for node B is then set based on $p_B|A$.
3. **Set Conditional Probabilities for Nodes C and D:**

- The switching probabilities for nodes C and D are determined similarly, taking into account the states of all preceding nodes (A and B for C, and A, B, and C for D). The CPT guides the probability settings for each conditional branch.
- The voltages for nodes C and D are set accordingly, ensuring that the network outputs values that follow the target PDF.

Conditional Probability Table (CPT): The overall conditional probability table of the 4 MTJs is summarized in Supplementary Table I, where $S(i, j)=\sum_{N=i}^{j} P(N)$. The respective driving voltages for each MTJ can be back-calculated using $V_{A/B/C/D}=\sigma_{A/B/C/D}^{-1}(P)$.

Supplementary Table I. Conditional Probability Table (CPT). P is the sampling probability and O is the outcome of the sampling. $S(i, j)=\sum_{N=i}^{j} P(N)$.

P_A	O_A	P_B	O_B	P_C	O_C	P_D	O_C
$\frac{S(8,15)}{S(0,15)}$	0	$\frac{S(4,7)}{S(0,7)}$	0	$\frac{S(2,3)}{S(0,3)}$	0	$\frac{P(1)}{S(0,1)}$	0
							1
					1	$\frac{P(3)}{S(2,3)}$	0
							1
			1	$\frac{S(6,7)}{S(4,7)}$	0	$\frac{P(5)}{S(4,5)}$	0
							1
					1	$\frac{P(7)}{S(6,7)}$	0
							1
	1	$\frac{S(12,15)}{S(8,15)}$	0	$\frac{S(10,11)}{S(8,11)}$	0	$\frac{P(9)}{S(8,9)}$	0
							1
					1	$\frac{P(11)}{S(10,11)}$	0
							1
			1	$\frac{S(14,15)}{S(12,15)}$	0	$\frac{P(13)}{S(12,13)}$	0
							1
					1	$\frac{P(15)}{S(14,15)}$	0
							1

Supplement III: Statistical evaluation of the TRNG-generated bitstreams

In this study, the MTJs used for TRNG were fabricated and tested using the same experimental protocols as those detailed in our recent publication[1]. In that work, we performed an extensive evaluation of the random bitstreams using NIST SP800-22 statistical test suite. The MTJ-based TRNG passed all tests with high confidence without requiring post-processing, thanks to the implementation of a hybrid control strategy that actively stabilizes switching probabilities across environmental and temporal variations. This demonstrates that the raw bitstreams generated by our MTJs already exhibit high-quality randomness and entropy, eliminating the need for whitening or post-processing in most use cases.

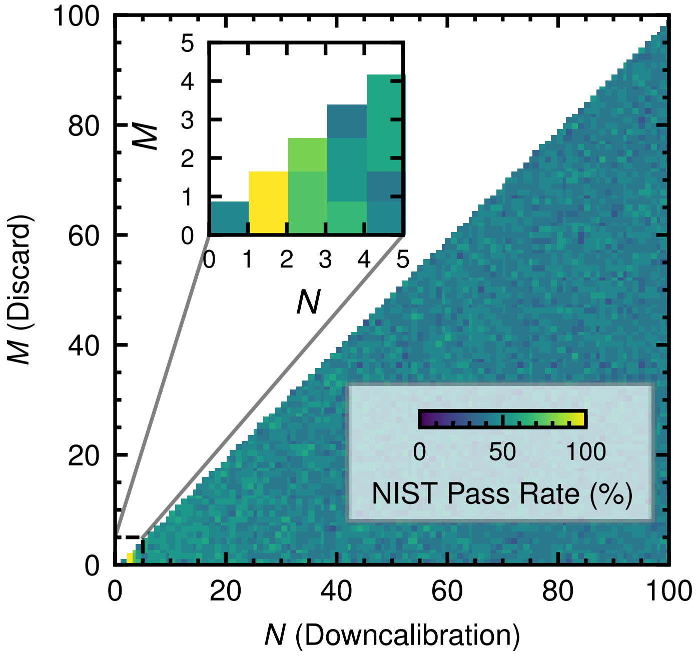

Figure S1. The MTJ-based TRNG passed all NIST tests without requiring post-processing, under Downcalibration-2 strategies. This calibration strategy to stabilize sampling probability and maintaining randomness can be found in Ref. [1].

Supplement V: Detailed derivation of the Probabilistic Greedy Algorithm for solving the Traveling Salesman Problem (TSP)

This supplement provides a comprehensive explanation of the probabilistic greedy algorithm used for solving the Traveling Salesman Problem (TSP). It elaborates on the theoretical convergence of the algorithm, the role of the temperature parameter $k_B T$, and the distinctions between this approach and the Boltzmann Machine (BM) algorithm. Although both algorithms share the characteristic of theoretical convergence, they differ in terms of how they handle the temperature parameter and the complexity associated with the number of simultaneously considered nodes.

Problem Definition and Background

The TSP is a fundamental combinatorial optimization problem that involves finding the shortest possible route for a salesman to visit a set of cities exactly once and then return to the starting city. The complexity of the problem increases exponentially with the number of cities, making exact solutions computationally infeasible for large instances. Various heuristic and probabilistic methods, such as simulated annealing, genetic algorithms, and greedy algorithms, have been developed to provide approximate solutions.

The classic greedy algorithm for solving the TSP selects the nearest unvisited city at each step. While this deterministic approach is computationally efficient, it often results in suboptimal solutions because it can become trapped in local minima. To address this limitation, the probabilistic greedy algorithm introduces a controlled degree of randomness into the city selection process, enabling the algorithm to escape local minima and explore a broader range of potential solutions.

Overview of the Probabilistic Greedy Algorithm

The probabilistic greedy algorithm modifies the traditional greedy approach by incorporating randomness into the selection of the next city. At each step, the probability of selecting a city is determined not solely by its distance from the current city but also by a temperature parameter $k_B T$, which adjusts the level of randomness in the selection process. The temperature parameter influences the balance between exploration (randomness) and exploitation (favoring shorter distances).

Probability Distribution for City Selection

The algorithm assigns a probability P_j to each unvisited city j, calculated as:

$$P_j = \frac{\exp(-d_{ij} / k_B T)}{\sum_{k \in U} \exp(-d_{ik} / k_B T)},$$

where:

- d_{ij} is the distance from the current city i to an unvisited city j,
- U is the set of all unvisited cities,
- $k_B T$ is the temperature parameter that determines the impact of distance on the selection probability.

Noticeably, there is a specialty for the case of the second to last city. Clearly, once the last second to last city is chosen, the last city is automatically determined. Supposing the last two cities are k and l, and the current city is m, therefore, the probability of the second to last city being k is determined as following.

$$P_{n-1+1}(k)=\frac{\exp\left(-\frac{d_{m\to k}+d_{k\to l}+d_{l\to 1}}{k_B T}\right)}{\exp\left(-\frac{d_{m\to k}+d_{k\to l}+d_{l\to 1}}{k_B T}\right)+\exp\left(-\frac{d_{m\to l}+d_{l\to k}+d_{k\to 1}}{k_B T}\right)}$$

Therefore, finding a complete route S returning to the starting city is the joint probability of P_j.

$$P(S)=\prod_{j=2}^{n}P_j \propto \exp\left(-\frac{d_{1\to 2}+d_{2\to 3}+\cdots+d_{n-1\to n}+d_{n\to 1}}{k_B T}\right)=\exp\left(-\frac{S}{k_B T}\right)$$

Here S is the total distance of the travelling loop.

The probability distribution P_j ensures that cities closer to the current city have a higher likelihood of being selected, but the influence of distance diminishes as $k_B T$ increases. The temperature parameter thus acts as a control knob:

- **Low $k_B T$**: When $k_B T \to 0$, the algorithm behaves like a traditional greedy algorithm, with the nearest city being almost always chosen. The selection process is nearly deterministic.
- **High $k_B T$**: When $k_B T \to \infty$, the selection probabilities become uniform across all unvisited cities, making the selection process entirely random.
- **Intermediate $k_B T$**: For values between these extremes, the algorithm allows some degree of randomness, helping it avoid local minima and explore different regions of the solution space.
- At a fixed T, the shortest loop with the smallest S thus has the highest probability $P(S)$ of being sampled in experiment, which ensures the convergence of this probabilistic greedy algorithm.

Finding the Optimal Temperature Parameter

A critical step in the probabilistic greedy algorithm is determining the appropriate value of $k_B T$. The goal is to find a temperature that provides a good balance between exploration and exploitation, allowing the algorithm to efficiently find high-quality solutions without excessively random behavior or determinism.

The optimal $k_B T$ is typically identified through empirical testing or a calibration phase in which the algorithm is run multiple times with different $k_B T$ values. Once a suitable $k_B T$ is found, it remains fixed for all subsequent iterations of the algorithm. This choice of a fixed temperature simplifies the implementation and avoids the need for its dynamic adjustments during the search process.

Convergence Mechanism with a Fixed Temperature

After identifying an optimal $k_B T$, the algorithm proceeds by repeatedly constructing routes using the fixed probability distribution. Each iteration of the algorithm represents an independent attempt to find a shorter path. The stochastic nature of city selection ensures that different paths may be explored in each iteration, even if the initial city remains the same.

1. **Fixed Temperature Advantage:** Keeping $k_B T$ fixed ensures that the probabilistic behavior is consistent across all iterations. Each run of the algorithm uses the same probability distribution for city selection, which helps in systematically exploring the solution space without the complications of dynamically adjusting the temperature.
2. **Iterative Refinement:** By repeatedly constructing tours with the fixed $k_B T$, the algorithm can find increasingly shorter paths over multiple iterations. Each run has the potential to discover a new local minimum or even the global minimum due to the probabilistic nature of the selection process. Over many repetitions, the algorithm converges towards an optimal or near-optimal solution.
3. **Sampling and Accumulation:** The algorithm effectively samples from the space of possible tours. As the number of iterations increases, the probability of finding a tour with a shorter total distance improves. This sampling-based approach ensures convergence over time by accumulating solutions that represent better approximations of the optimal path.

Theoretical Convergence Analysis

The theoretical basis for the convergence of the probabilistic greedy algorithm lies in its ability to explore many possible solutions due to the probabilistic selection of cities. Since the algorithm can, in principle, sample any possible tour (given enough iterations), the probability of finding the optimal solution approaches one as the number of iterations increases.

The algorithm's convergence rate depends on:

- **The quality of the chosen $k_B T$:** A well-selected temperature increases the likelihood of finding high-quality solutions in fewer iterations by balancing randomness and deterministic behavior.
- **The number of iterations:** More iterations lead to a higher probability of discovering shorter paths, as the algorithm can explore more regions of the search space.

Differences from the Boltzmann Machine (BM) Algorithm

While the probabilistic greedy algorithm and the Boltzmann Machine (BM) algorithm share similarities in using a temperature parameter to control randomness, they differ significantly in how they treat the temperature parameter as well as the number of nodes considered simultaneously:

1. **Fixed vs. Annealing Temperature:** In the probabilistic greedy algorithm, once an optimal $k_B T$ is found, it remains unchanged. The algorithm does not involve an annealing process where the temperature is gradually decreased over time. This contrasts with the BM algorithm, which typically employs an annealing process, starting with a high temperature and gradually lowering it to reduce randomness.
2. **Node Consideration and Entanglement Complexity:** The probabilistic greedy algorithm evaluates one decision at a time, selecting the next one from the unvisited cities based on the current state and distances. In other words, all the remaining cities are evaluated before a real sampling is drawn. In contrast, the BM algorithm with n^2 binary variables for a n-city TSP adopts the Gibbs sampling protocol, that is, updating only one variable in the n^2 nodes and keeping all other variables fixed. This locality of the sampling protocol therefore limits the entanglement complexity of the BM algorithm, which accounts for the better performance of the Probabilistic Greedy Algorithm than the BM.

Practical Implications of the Fixed Temperature Approach

The choice of using a fixed $k_B T$ has several advantages for practical applications:

- **Simplified Algorithm Design:** Keeping the temperature constant after finding an optimal value simplifies the algorithm's design and implementation. There is no need for a complex annealing schedule or adaptive temperature control.
- **Parallelization Opportunities:** Since each iteration is independent of the others, the algorithm can be easily parallelized, with multiple runs executed simultaneously to

explore different regions of the search space. This parallelism can significantly speed up the convergence process.

- **Suitable for Resource-Constrained Environments:** The fixed temperature approach reduces computational overhead compared to methods that require dynamic adjustments. It is particularly useful when computational resources are limited and when rapid convergence is desired.

Convergence Characteristics and Limitations

While the probabilistic greedy algorithm with a fixed temperature parameter has desirable convergence properties, it also has limitations:

- **Dependence on the Initial Selection of $k_B T$:** The algorithm's performance is highly dependent on finding a suitable $k_B T$. If the chosen temperature does not strike an appropriate balance, the algorithm may either get trapped in local minima (if $k_B T$ is too low) or fail to refine solutions effectively (if $k_B T$ is too high).
- **Lack of Global Consideration:** Since decisions are made sequentially, the algorithm does not account for the overall tour configuration at each step. This limitation contrasts with algorithms like the BM that consider the entire configuration simultaneously, potentially leading to better results for highly complex instances.

The use of the probabilistic greedy algorithm for solving the TSP provides a practical and flexible approach by introducing a controlled degree of randomness into the selection process. By fixing the temperature parameter $k_B T$ after an initial calibration and performing repeated trials, the algorithm achieves convergence through iterative refinement, accumulating increasingly better solutions. Its simplicity, suitability for parallel execution, and ability to maintain theoretical convergence make it an effective choice for solving large-scale TSP instances where traditional greedy algorithms and exhaustive methods may fall short.

The differences in handling temperature and node consideration compared to the Boltzmann Machine algorithm highlight the trade-offs in terms of entanglement complexity and computational requirements. While both approaches can theoretically converge to the optimal solution, the probabilistic greedy algorithm provides a simpler and more resource-efficient path to high-quality solutions for practical TSP problems.

To further enhance the clarity and intuitiveness of this mechanism, we provide an additional explanatory schematic (Figure S2), which illustrates how different temperature values ($k_B T$) alter the probabilistic decision-making process in our algorithm. At low temperature, the city with the shortest distance is deterministically chosen, mimicking a classic greedy algorithm. At high temperature, cities are selected with nearly equal probability, akin to random exploration.

At intermediate values, the selection probability is smoothly distributed following a Boltzmann distribution, striking a balance between exploration and exploitation.

This step-by-step probabilistic sampling is directly driven by our MTJ-based TRNGs, whose PDF can be dynamically updated to reflect the current decision state. The hardware block shown in Figure S2 (middle-bottom) highlights the compact structure needed for such TRNG-based selection: a small number of MTJs ($\log_2 N$ for N cities) suffices to implement arbitrary probability distributions at each step, thereby greatly reducing hardware complexity compared to conventional Ising-based solvers requiring $O(N^2)$ coupling nodes. This clear physical-to-algorithmic linkage — from MTJ voltage to switching probability to next-city selection — underpins the novelty and efficiency of our probabilistic greedy optimization strategy.

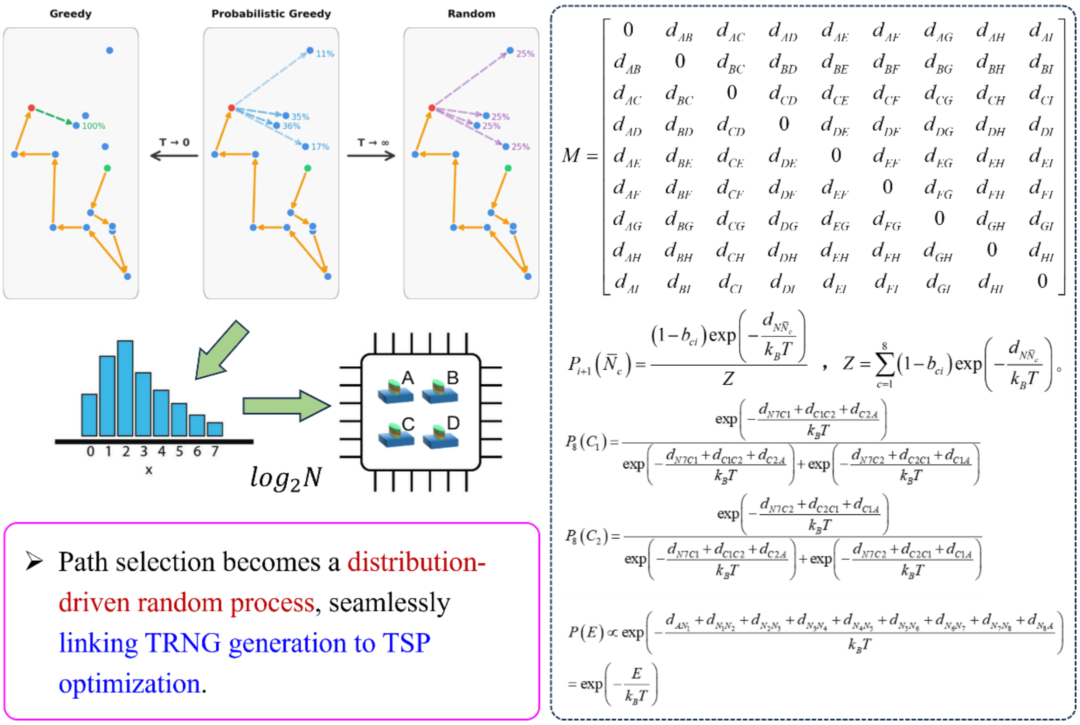

Figure S2. Schematic illustration of the decision-making process in the probabilistic greedy algorithm for solving TSP. Top: The influence of temperature (T) on city selection probabilities — from deterministic ($T\rightarrow 0$), to balanced probabilistic (intermediate T), to fully random ($T\rightarrow\infty$). Middle: A dynamically updated probability distribution is used to sample the next city. Bottom: This distribution is realized in hardware using MTJ-based TRNG arrays capable of generating binary outcomes according to any desired PDF, with a logic resolution of $\log_2 N$ MTJs for N cities. The overall system links real-time TRNG outputs to Boltzmann-distributed decision probabilities, efficiently guiding the optimization trajectory.

Supplement VI: Adaptation of the probabilistic greedy framework to graph coloring

We focused on the TSP as a representative NP-hard problem to demonstrate the efficacy of our framework, but the underlying principles are indeed applicable to a wide range of combinatorial optimization tasks. The key lies in formulating each problem's Loss (or Cost) function and incorporating it into the probabilistic decision-making process. For example, in graph coloring, the algorithm would probabilistically select how to color each node to minimize the number of conflicts, while in scheduling, it would seek to reduce overall makespan or resource contention. The "temperature" parameter would again play a similar role in balancing exploration and exploitation: at low temperature, the algorithm favors local minima; at high temperature, it explores more globally. Although the specific probability distribution shaping may need fine-tuning to reflect the constraints or cost landscape of each problem, the overarching method — leveraging MTJ-generated randomness within a probabilistic greedy framework — remains largely intact. Therefore, we anticipate that only minor modifications, such as redefining the cost function and adjusting the temperature schedule, would be required and enough to generalize our approach to other NP-hard scenarios.

Here we can easily adopt the idea of probabilistic greedy algorithm into the graph coloring problem with the basic equations as follows. The graph coloring problem requires assigning colors to the vertices of a graph $G(V, E)$, such that no two adjacent vertices share the same color, while minimizing the total number of colors used. To adapt our MTJ-assisted probabilistic greedy framework, we define a probabilistic selection process for color assignment at each step, guided by a dynamically adjusted probability distribution based on a cost function. Let us define:

- $V = \{v_1, v_2, \ldots, v_n\}$: the set of vertices,
- $C = \{c_1, c_2, \ldots, c_k\}$: the set of available colors (with k large enough to allow a feasible coloring),
- conflict (v_i, c_j): the number of adjacent vertices of v_i already assigned color c_j.

For each vertex v_i, we define the cost of assigning color c_j as:

$$E_{ij} = \lambda \cdot \mathrm{conflict}(v_i, c_j)$$

where $\lambda > 0$ is a penalty weight that amplifies the cost of conflicts.

The probability of assigning color c_j to vertex v_i is then determined by the Boltzmann distribution:

$$P_{ij} = \frac{e^{-E_{ij}/(k_{\mathrm{B}}T)}}{\sum_{l=1}^{k} e^{-E_{il}/(k_{\mathrm{B}}T)}}$$

This formulation ensures that color assignments with fewer conflicts are more probable, especially at lower temperature T, while allowing more exploration at higher T.

The coloring process proceeds as follows:

1. Initialize all vertex colors to unassigned.
2. For each uncolored vertex v_i, compute P_{ij} for all available colors $c_j \in C$ based on the current partial coloring.
3. Use MTJ-based TRNG and PDF control to sample from P_{ij} and assign color c_j to v_i.
4. Repeat steps 2 – 3 until all vertices are colored.

Optionally, the total number of used colors can be minimized by starting from a conservative upper bound k, and gradually reducing it while checking feasibility.

This probabilistic greedy strategy preserves the same core elements from our TSP implementation:

- The stochastic nature is governed by tunable $k_B T$,
- MTJ-generated Bernoulli bits are used to construct arbitrary PDFs for decision-making,
- The decision process is localized and sequential, making it naturally scalable.

This adaptation highlights the generality of our method: by redefining the cost function E and updating the probability calculation, the same hardware-friendly framework can be extended to a wide class of combinatorial optimization problems.

Example: End-to-end simulation on a 5-node cycle

To illustrate the feasibility of the proposed probabilistic greedy framework beyond TSP, we provide a toy example of graph coloring on a 5-node cycle C_5. The vertex set is $V = \{v_1, \ldots, v_5\}$ and the edge set is $E = \{(v_1, v_2), (v_2, v_3), (v_3, v_4), (v_4, v_5), (v_5, v_1)\}$. Three colors $C = \{1, 2, 3\}$ are available, which suffices since the chromatic number of C_5 is 3.

The conflict cost for assigning color c to vertex v is defined as

$$E(v,c) = \lambda \cdot \#\{\text{neighbors of } v \text{ already assigned color } c\}, \lambda = 1,$$

and the sampling probability follows the Boltzmann distribution

$$P(v,c) = \frac{\exp\left(-\frac{E(v,c)}{T}\right)}{\sum_{c' \in C} \exp\left(-\frac{E(v,c')}{T}\right)},$$

with temperature T = 0.5. In hardware, each sampling step requires $\lceil log_2|C|\rceil$ MTJs to generate a $|C|$-ary outcome.

We sequentially color vertices $v_1 \rightarrow v_2 \rightarrow v_3 \rightarrow v_4 \rightarrow v_5$. The detailed steps are summarized in Table S1.

**Step**	**Current vertex**	**Neighbor colors**	**Weights [w_1, w_2, w_3]**	**Normalized $P(1)$, $P(2)$, $P(3)$**	**Sampled color**	**Partial coloring**
1	v_1	none	[1, 1, 1]	[0.333, 0.333, 0.333]	1	v_1 = 1
2	v_2	v_1 = 1	[0.135, 1, 1]	[0.063, 0.469, 0.469]	2	v_1 = 1, v_2 = 2
3	v_3	v_2 = 2	[1, 0.135, 1]	[0.469, 0.063, 0.469]	3	v_1 = 1, v_2 = 2, v_3 = 3
4	v_4	v_3 = 3	[1, 1, 0.135]	[0.469, 0.469, 0.063]	1	v_1 = 1, v_2 = 2, v_3 = 3, v_4 = 1
5	v_5	v_1 = 1, v_4 = 1	[0.018, 1, 1]	[0.009, 0.496, 0.496]	2	v_1 = 1, v_2 = 2, v_3 = 3, v_4 = 1, v_5 = 2

This run produces a valid 3-coloring (1, 2, 3, 1, 2) with no conflicts. Since the algorithm is stochastic, repeating the process yields different but typically valid solutions. At lower temperature, the distribution is sharply biased towards conflict-free choices, while at higher temperature the algorithm explores more broadly.

Pseudocode:

```
Input: Graph G = (V, E), color set C, parameters λ, T
Initialize all colors[v] ← UNCOLORED
for v in visit_order(V) do
    for c in C do
        conflicts ← #(u in N(v) with colors[u] = c)
        E[c] ← λ * conflicts
        w[c] ← exp(−E[c]/T)
    end for
    P ← w / sum(w)
    colors[v] ← SampleFrom(P)   // implemented by MTJ-TRNG
end for
return colors
```

This example demonstrates that the probabilistic greedy framework can be directly applied to graph coloring, with complexity O($|V|\cdot|C|$) auxiliary memory, and only $\lceil log_2|C|\rceil$ MTJs required for each sampling step.

Reference

1. Zhang, R. *et al.* Drift-resilient magnetic-tunnel-junction random-number generator via hybrid control strategies. *Phys. Rev. Appl.* **23**, 054073 (2025).
